# Immunotherapeutic approaches in EBV-associated nasopharyngeal carcinoma

**DOI:** 10.3389/fimmu.2022.1079515

**Published:** 2023-01-11

**Authors:** Wenting Li, Xiaobing Duan, Xingxing Chen, Meixiao Zhan, Haichuan Peng, Ya Meng, Xiaobin Li, Xian-Yang Li, Guofu Pang, Xiaohui Dou

**Affiliations:** ^1^ Guangdong Provincial Key Laboratory of Tumor Interventional Diagnosis and Treatment, Zhuhai People’s Hospital (Zhuhai Hospital Affiliated with Jinan University), Zhuhai, China; ^2^ Department of Urology, Zhuhai People’s Hospital (Zhuhai Hospital Affiliated with Jinan University), Zhuhai, China; ^3^ Faculty of Health Sciences, University of Macau, Macau, Macau SAR, China; ^4^ Department of R&D, OriCell Therapeutics Co. Ltd, Pudong, Shanghai, China; ^5^ Health Management Center, Zhuhai People’s Hospital (Zhuhai Hospital Affiliated with Jinan University), Zhuhai, China

**Keywords:** Epstein-Barr virus, EBV-associated cancer, EBV-directed vaccination, adoptive cell therapy, TCR-T therapy, immune checkpoint inhibitors

## Abstract

Epstein–Barr virus (EBV) was the first tumor virus in humans. Nasopharyngeal carcinoma (NPC) accounts for approximately 60% of the 200,000 new tumor cases caused by EBV infection worldwide each year. NPC has an insidious onset and is highly malignant, with more than 70% of patients having intermediate to advanced disease at the time of initial diagnosis, and is strongly implicated in epithelial cancers as well as malignant lymphoid and natural killer/T cell lymphomas. Over 90% of patients with confirmed undifferentiated NPC are infected with EBV. In recent decades, much progress has been made in understanding the molecular mechanisms of NPC and developing therapeutic approaches. Radiotherapy and chemotherapy are the main treatment options for NPC; however, they have a limited efficacy in patients with locally advanced or distant metastatic tumors. Tumor immunotherapy, including vaccination, adoptive cell therapy, and immune checkpoint blockade, represents a promising therapeutic approach for NPC. Significant breakthroughs have recently been made in the application of immunotherapy for patients with recurrent or metastatic NPC (RM-NPC), indicating a broad prospect for NPC immunotherapy. Here, we review important research findings regarding immunotherapy for NPC patients and provide insights for future research.

## 1 Introduction

**Figure 1:**
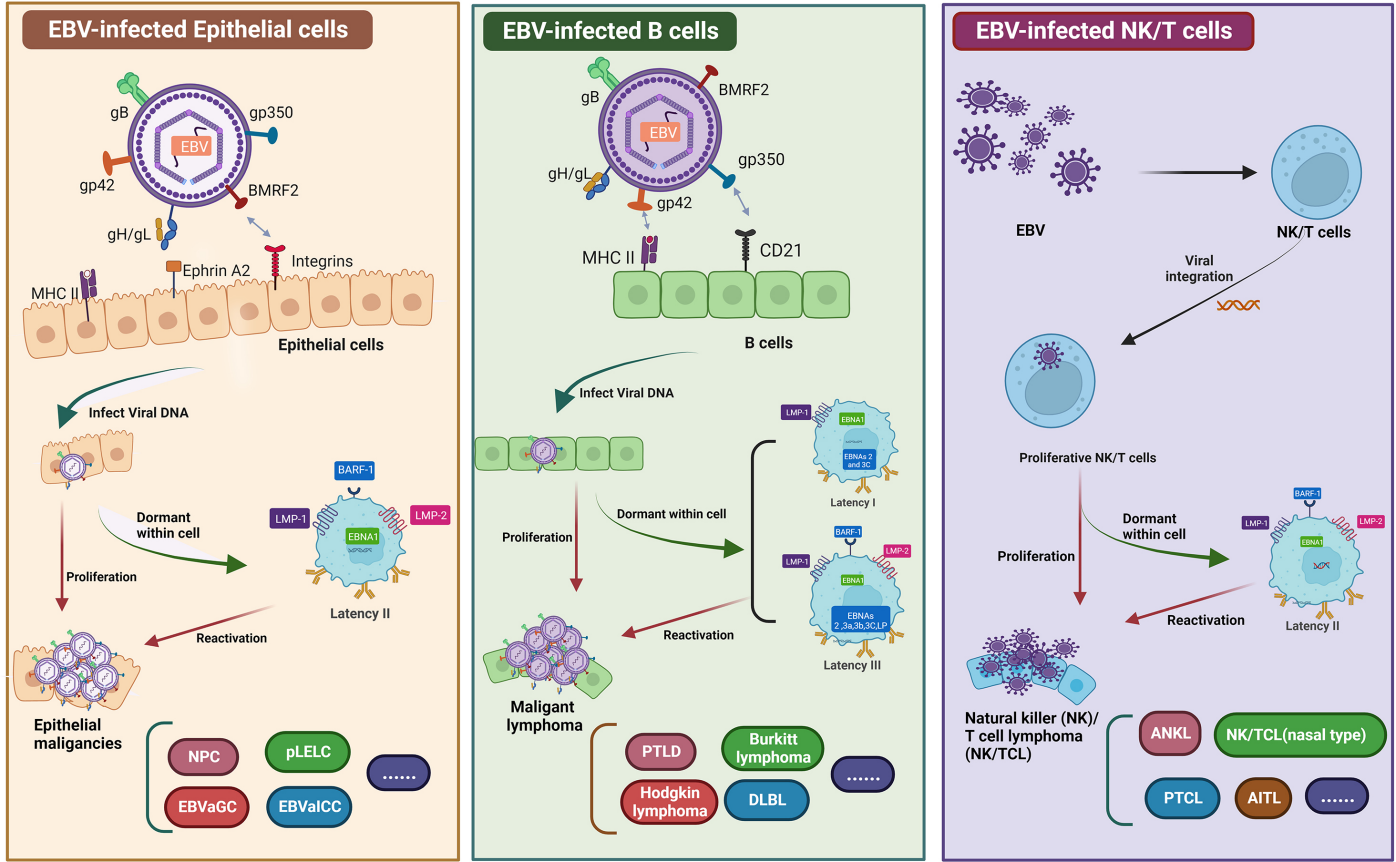
Graphical summary of EBV infected epithelial cells, B cells, and NK/T cell. EBV primarily infects epithelial and B cells. Infection with EBV may cause many human cancers, including epithelial cancers such as EBVaGC, NPC, EBVaICC, and pLELC. EBV-infected B cells can lead to malignant lymphomas, such as DLBL, PTLD, and BL. Similarly, EBV infects NK/T cells to form NK/T cell lymphomas. EBV establishes a persistent latent infection in malignant epithelial cells (known as latency I, latency II, and latency III). All EBV-associated epithelial cancers express a latency II program (8–12). Two therapeutic vaccines have been investigated for NPC, namely, peptide-based vaccines and DC vaccines. EBV requires a variety of envelope proteins to enter cells. The membrane proteins gp350, gH/gL, gB, and gp42 are required for B cell infection, whereas BMFR2, gH/gL, and gB are needed for epithelial cell infection. Another vaccine under study mainly uses LMP1, LMP2, EBNA1 and EBNA3 as target antigens to construct viral vaccines by single or multiple protein combinations. Figure was created with BioRender.

Approximately 95% of the global population are EBV asymptomatic carriers (1, 2). EBV primarily infects epithelial and B cells. Infection with EBV may cause many human cancers, including malignant lymphoid and epithelial cancers such as NPC, primary pulmonary lymphoepithelioma-like carcinoma (PLELC), EBV-associated intrahepatic cholangiocarcinoma, and EBV-associated gastric carcinoma (EBVaGC) [ (3–7), **Figure 1**]. NPC and EBVaGC are the two most common EBV-associated epithelial malignancies that account for 80% of these tumors. Over 90% of patients with confirmed undifferentiated NPC are infected with EBV (2, 13, 14). According to the last two global cancer statistical surveys from the International Agency for Research on Cancer, in 2018 and 2020, there were 129,079 and 133,354 new NPC cases in the world, and 72,987 and 80,008 NPC-associated deaths worldwide, respectively (15, 16). NPC is relatively rare compared to other cancers, accounting for only 0.7% of total cancers diagnosed each year. The geographical distribution of NPC is uneven, as approximately 80% of patients are from China and Southeast Asia (17, 18). Although both gene and lifestyle affect NPC incidence, EBV infection is particularly closely related to NPC, making EBV a unique target for tumor immunotherapy.

**Figure 2:**
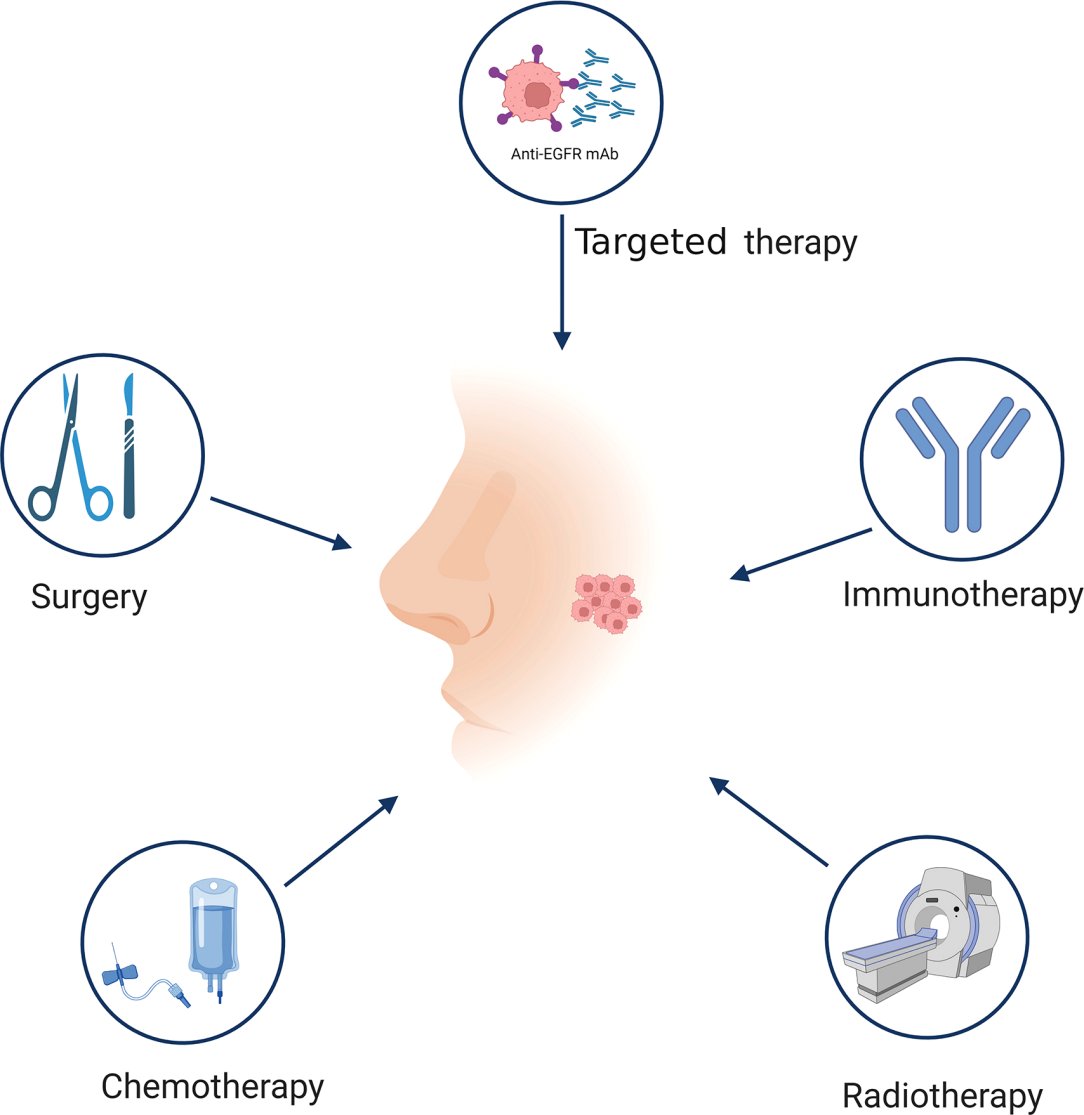
Treatment of NPC. Different stages of NPC have different treatment methods. The traditional treatment of NPC includes surgery, radiotherapy, chemotherapy, and targeted therapy. Immunotherapy is a promising therapeutic approach for NPC treatment. Figure was created with BioRender.

In accordance with the International Union Against Cancer staging system, NPC are graded according to several stages (I–IVB) (19–21). NPC treatment options vary depending on the stage. Conventional therapy for NPC includes surgery, radiotherapy, and chemotherapy (**Figure 2**). Owing to the deep tumor localization and complex anatomical structure of the tumor site, surgical options are limited. However, NPC is a highly radiosensitive and chemosensitive tumor; therefore, radiotherapy and chemotherapy alone or in combination are the primary treatments for patients with stage I/II NPC (22, 23). Although combined chemoradiotherapy has good prognosis (85–90% survival over 5 years), its efficacy is nonetheless limited, and approximately 8–10% of patients experience recurrence or metastasis (24–26). Platinum plus multidrug therapy is the preferred treatment for RM-NPC patients, however, the eventually developing resistance is a major barrier to successful treatment (27). Concurrent chemoradiotherapy is of great importance for improving treatment outcomes in locally advanced NPC but often leads to complications, such as xerostomia, trismus, and secondary tumors, which seriously impact patients’ quality of life (28–32). Concurrent chemoradiotherapy based on cisplatin is the standard treatment for patients with locally advanced NPC (33), and more than 50% of NPC patients are initially confirmed as advanced stage (34, 35). Therefore, the development of new strategies that not only prolong the disease-free survival of patients but also reduce treatment-related complications and adverse events is of critical clinical importance.

At present, the clinical efficacy of radiotherapy or chemotherapy alone for the patients with RM-NPC is suboptimal, and it may be improved by the use of immunotherapy. The dense matrix infiltration by immune cells and EBV antigen expression in NPC patients are the main research targets for immunotherapy (36, 37). The primary strategies for immunotherapy are vaccination, adoptive cell therapy (ACT), and immune checkpoint blockade. Here, we review important research advances in the field of NPC immunotherapy, hoping to provide insights for future studies.

## 2 EBV-­directed vaccination

**Figure 3:**
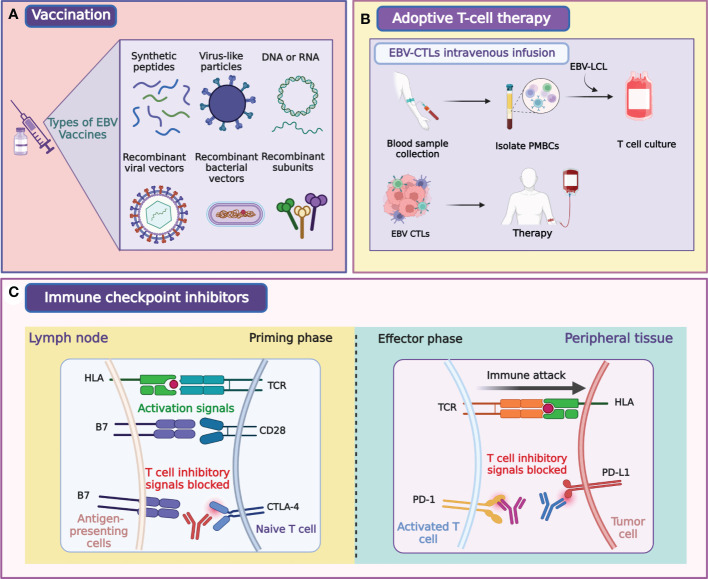
Illustration of major modalities of tumor immunotherapy for NPC. Primary strategies of tumor immunotherapy for NPC include EBV-directed vaccination **(A)**, EBV-CTLs intravenous infusion **(B)**, and immune checkpoint blockade **(C)**. Figure was created with BioRender.

Infection with EBV primarily leads to epithelial and B cell malignancies, including NPC, infectious mononucleosis (IM), and Burkitt’s lymphoma (38–42). Concurrent chemoradiotherapy, induction chemotherapy, and adjuvant chemotherapy are the three main therapies for NPC patients that have good curative effects. However, the emergence of drug resistance and adverse events limit the application of chemotherapy for NPC (29). Sources of EBV infection are widespread, and transmission routes are difficult to cut off, similar to those of human papillomavirus and hepatitis B virus (43, 44). Vaccination is the most effective treatment to prevent infection with EBV and the most cost-effective way to treat IM and EBV-associated diseases, such as multiple sclerosis [(45), **Figure 3A**]. An effective EBV vaccine will make a great impact on public health and the economy. The use of EBV preventive vaccines is intended to prevent EBV infection of target cells by eliciting neutralizing antibodies (43–45). Two types of vaccines have been investigated for NPC treatment, peptide and viral vaccines. Peptide vaccines primarily focus on gp350, which stimulates the body to produce neutralizing antibodies to block the virus infection pathway after inoculation. Other antiviral vaccines currently being studied use LMP1, LMP2, EBNA1, and EBNA3 as target antigens or their combinations (46–50).

EBV needs to pass through a variety of membrane proteins to enter the cytoplasm of the epithelial, B cells, and NK/T cells. The infection of B cells by EBV requires five membrane proteins (gp350, gH, gL, gB, and gp42), whereas the infection of epithelial cells by EBV needs four membrane proteins (BMFR2, gH, gL, and gB). These proteins are expressed on EBV and could be good targets for EBV-preventive vaccine (35, 51–56). Studies on vaccines for EBV-related diseases were began in the 1980s, with approximately half the studies focusing on the EBV protein gp350 (also known as gp340) that binds to CD21/CD35 of B cells [ (57, 58), **Table 1**]. Early studies demonstrated that the gp350-containing vaccine impacts the infection process. Non-recombinant gp340 vaccine was first shown to work against EBV-induced lymphoma in an animal trial in the 1980s (70). In the early 1990s, the first clinical trial of a vaccine containing gp350 were conducted in China. Subjects developed corresponding antibodies after receiving the vaccine; however, this vaccine was not further developed (59). Two clinical trials of the vaccine containing recombinant gp350 showed considerable efficacy (mean response rate, 78.0%) in preventing IM caused by EBV infection but not the asymptomatic EBV infection (60, 61). This was most likely because gp350-induced antibodies did not protect epithelial cells from EBV infection. Therefore, it is important to identify new target antigens to develop an effective EBV-preventive vaccine. EBV glycoproteins gH/gL and gB co-mediate the fusion step of EBV into B cells or epithelium. Inoculation with vaccines containing these proteins induced the development of antibodies that could broadly protect against EBV infection (71, 72). The immunogens of proteins expressed or polymerized by multimers are relatively stronger than those of single protein polymers (73–75). Cui et al. compared the vaccines based on the tetrameric and monomeric isoforms of the gp350 protein in ovarian cells and found that the tetrameric gp350 had markedly higher immunogenicity than its monomeric counterpart. These data showed that the application of the tetrameric gp350^1–470^ and EBV protein multimerization, in general, may facilitate the development of a potent prophylactic EBV vaccine (76). According to two reports published in 2016 and 2021, serum neutralization titers of antibodies raised against EBV gp350 monomer were lower than those induced by three dimer and trimer gH/gL, trimer gB, or four gp350^1–470^ polymers (77, 78). This may be attributed to the key role of gH/gL and gB in the fusion and entry of EBV into B cells or epithelium. Therefore, compared with gp350 alone, EBV gH/gL and gB may be the better targets for EBV-preventive vaccine, and gp350 binding with gH/gL, gB may yield a stronger EBV-preventive vaccine. In 2021, the gH/gL-specific antibody 1D8 was isolated from EBV-infected individuals to target EBV-vulnerable sites (79). 1D8 binds to the critical fragile interfaces of viral gH/gL proteins, disrupting the gH/gL-mediated fusion of EBV with the membrane of target cells. The discovery of antibodies against new EBV targets is therefore highly warranted. Our team comprehensively described the response between T cells and full-length gB. *In vitro*, gB-specific CD8^+^ T cells were found to inhibit the transformation of B cells; available gB epitopes, including gB D-II and D-IV, were identified; and two specific gB antibodies (3A3 and 3A5) were isolated. The two antibodies were identified to target and neutralize EBV-infected B cells and epithelial cells. These studies indicate that accurate localization of gB T cell epitopes is beneficial to the development of gB subunit vaccines and immune surveillance and that gB D-II and D-IV are promising targets for the development of EBV vaccines (58, 80).

**Table 1:** Illustration of completed and documented EBV vaccine trials.

Vaccine	Year	Cohort size	Clinical outcome
**Live recombinant virus gp350 vaccinia**	1995	9	Vaccination boosted EBV-neutralizing antibody titers; no vaccine efficacy (59).
**Recombinant subunit gp350 EBV vaccine purified from Chinese hamster ovary cells**	2007	148	One serious adverse event occurred which was considered to be of suspected relationship to vaccination; no vaccine efficacy (60).
**Recombinant gp350 vaccine**	2007	181	Recombinant gp350 showed significant efficacy (mean response rate, 78.0%) in preventing infectious mononucleosis caused by EBV infection, but not in preventing asymptomatic EBV infection (61).
**EBV peptide vaccine**	2008	14	The vaccine was well tolerated,and 1/2 placebo vaccines who acquired EBV developed infectious mononucleosis. Single-epitope vaccination did not predispose individuals to disease, nor did it significantly influence development of a normal repertoire of EBV specific CD8(+) T-cell responses following seroconversion (62).
**Recombinant gp350 vaccine**	2009	16	The vaccine was immunogenic but a prolonged vaccine schedule up to time of transplantation or improved adjuvants are required in future trials to reduce post (63).
**Adenovirus ΔLMP1–LMP2** **transduced DC vaccine**	2012	16	No increase detected in the frequency of peripheral LMP1/2-specific T cells (64).
**AdE1-LMPpoly vaccine**	2012	24	Adoptive immunotherapy with AdE1-LMPpoly vaccine is safe and well tolerated and may offer clinical benefit to patients with NPC (65).
**EBV-specific HLA-A2-restricted** DC vaccine	2013	16	9 patients responded to LMP2A peptides,and serum EBV-DNA level significantly decreased; The EBV-specific HLA-A2-restricted DC vaccination is a promising treatment for EBV-related NPCs (66).
**Recombinant vaccinia virus, MVA-EL, which encodes an EBNA1/LMP2 fusion protein**	2013	18	T-cell responses to EBNA1 and/or LMP2 increased in 15 patients; MVA-EL was both safe and immunogenic (67).
**MVA-EL**	2014	16	T-cell responses to EBNA1 and/or LMP2 increased in 8 patients; MVA-EL was safeand immunogenic across diverse ethnicities and thus suitable for use in trials against different EBV-positive cancers globally (68).
**Adenoviral vaccine of EBV-LMP2 (rAd5-EBV-LMP2)**	2016	24	Proportion of CD3^+^ CD4^+^ cells in peripheral blood significantly increased; The rA5-EBV-LMP2 vaccine was safe and well-tolerated, but has no vaccine efficacy (69).

Despite numerous attempts to produce vaccines against EBV, none has been licensed to date. Virus-like particles (VLPs) may be promising vaccine candidates. The VLP-based vaccine against hepatitis B and HPV have been widely used in clinical practice, suggesting that a similar approach may be fruitful for an effective vaccine against EBV (79, 81–83). The first complete EBV VLPs had knockouts of the potential cancer-causing genes and repetitive sequences in the EBV terminal, such as LMP1 and BZLF1. These EBV VLPs elicited EBV-specific responses in mice after immunization (45, 84). A study constructed an EBV VLP-based vaccine on the basis of a novel Newcastle disease virus (NDV). The platform, named EBVgp350/220-F, was formed by the fusion of EBVgp350/220 with the NDV fusion (F) protein (85). *In vivo* experiments, EBVgp350/220-F VLPs induced a high and persistent neutralizing antibody response in mice (85). At present, the platform of NDV VLP is applied to developing gH/gL-EBNA1 and gB/LMP2 VLPs. LMP1, LMP2, EBNA1, and EBNA3, alone or in combinations, are primarily used as target antigens for anti-EBV vaccines because of their important roles in mediating EBV infection and subsequent malignant transformation of infected cells (50, 86–88). A clinical trial performed in 2002 showed that immunization with EBV peptide-pulsed DCs induces tumor disappearance in patients with EBV-induced NPC (89). Epitope-specific CD8^+^ T cell responses were induced or enhanced in 9 out of 16 subjects (56%) in that trial. In another study, 16 patients with HLA-A2-positive stage II/III NPC were administered autologous DCs containing the HLA-A2-restricted LMP2A peptide, and in nine of them, the responses of circulating LMP2-specific T cells were improved and serum EBV DNA levels were modestly reduced (66). There was a study assessed the efficacy of Ad-ΔLMP1-LMP2 transduced DCs vaccine in total 12 patients with NPC, this vaccine increased the activity of LMP1/2-specific T cells *in vitro* (64). Furthermore, in nine out of these 12 patients, this vaccine caused late-onset hypersensitivity reactions (64). Thus, although the clinical efficacy of DC-based vaccines in these clinical trials was mixed, the safety and tolerability of EBV vaccines in NPC patients were consistently demonstrated.

Recently, recombinant viral vector vaccines, such as the one based on modified vaccinia Ankara (MVA), have been created (90, 91). One study showed that an MVA fusion protein could effectively reactivate CD4^+^ memory T cell responses *in vitro*, and the MVA fusion protein comprised both the carboxyl terminus of EBNA1 and full-length LMP2(MVA-EL) (92). The first human MVA-EL vaccine trial involving 18 NPC patients was conducted in Hong Kong (67). Responses of T cells to vaccine antigens were enhanced in fifteen out of the 18 subjects in that study; therefore, MVA-El was safe and immunogenic. A separate phase I study of the MVA-EL vaccine in the patients with NPC was conducted in the United Kingdom, which demonstrated increased CD4^+^ and CD8^+^ T cell responses to antigens in eight out of 14 patients (68). Another two clinical trials with this vaccine, NCT01800071 and NCT01094405, are in progress.

Recently, an mRNA vaccine has been successfully used against SARS-CoV-2, and a synthetic mRNA vaccine against EBV is currently in a clinical trial (phase I, NCT05164094). Although mRNA vaccines have many advantages than conventional vaccines, there are also potential challenges.

Thus, clinical trials demonstrated that vaccines against EBV have substantial clinical value. Nonetheless, they also have some limitations: DC-based vaccines have few targets and high preparation cost, whereas although recombinant virus vectors have broad epitopes, their immune function may be reduced after repeated immunizations. It should be noted that different administration routes should be systematically examined to facilitate EBV vaccine delivery. Future vaccines containing EBNA1, LMP1/2, gp350, gH/gL, and gB are promising directions for EBV vaccine research.

## 3 Adoptive cell therapy

In recent years, ACT has become a powerful strategy in the treatment of human cancers, especially some blood malignancies. However, extending these therapies to solid cancers such as NPC remains problematic. Here, we discuss recent developments in several ACT approaches.

### 3.1 EBV-specific cytotoxic T lymphocyte therapy

EBV-associated posttransplant lymphoproliferative disorders (PTLDs) are common complications in solid organ and bone marrow transplant recipients (93–96). PTLDs are related to the massive proliferation of EBV-B cells, cytotoxic T lymphocytes (CTLs) control its expansion in immunologically active individuals (97). Many clinical trials showed that ACT with EBV-specific CTLs prevented and treated PTLDs in patients with hematopoietic stem cell transplantation (98–102). EBV-specific CTLs also effectively treated PTLDs in patients with solid organ transplants that were on high immunosuppression regimens (93–95, 103, 104). These results have broadened the application of ACT in EBV-associated cancers, and both EBNA1 and LMP1 were the targets for the amplification of EBV-specific CTLs in the majority of studies (65, 105–111). The clinical ACT approach used for NPC treatment involves lymphoblastoid cell line-generated EBV-specific T cells (**Figure 3B**; **Table 2**). A clinical trial using EBV-specific CTLs to treat NPC patients showed that the content of EBV DNA in plasma was decreased to an undetectable level in all subjects (112). A clinical study published in 2004 showed that EBV-specific CTLs can be effectively used to treat NPC patients, and proved that EBV-specific CTLs with anticancer properties detected *in vitro* could increase the LMP2-specific immune response (113). Another study that used EBV-specific CTLs to treat NPC showed that disease progression was controlled in 6 of 10 patients. This trial also demonstrated that EBV-targeted autologous CTL therapy was safe and induced LMP2-specific immune activity that controlled the disease progression in stage IV NPC resistant to conventional therapy (107). Two clinical trials by Straathof et al. further proved that this treatment was safe and performed a activity for anti-tumors (108, 114).

**Table 2:** Completed clinical trials of EBV-CTLs in NPC.

Treatment	Year	Cohort size	Clinical outcome
**Autologous EBV-CTLs**	2001	4	The treatment was safe and unaccompanied by inflammatory or other complications, but whether it improved tumor control could not be discerned from the large tumor bulk. 3/4: EBV burden decrease; 3/4: die of PD (9–21 months after EBV-CTLs) (112).
**EBV-specific CTLs**	2004	1	Adoptive transfer of allogeneic Epstein-Barr virus (EBV)-specific cytotoxic T cells with *in vitro* antitumor activity boosts LMP2-specific immune response in a patient with EBV-related NPC;Preliminary data obtained in this patient indicate that EBV-specific CTLs are safe, may exert specific killing of NPC tumor cells *in vitro*, and induce antitumor effect *in vivo* (113).
**Autologous EBV-specific CTLs (LCL-stimulated CTL with low-dose IL-2)**	2005	10	Cell therapy with EBV-targeted autologous CTLs is safe, induces LMP-2-specific immunologic responses, and is associated with objective responses and control of disease progression in patients with stage IV NPC resistant to conventional treatments (107).
**Autologous EBV-specific CTLs**	2005	10	Administration of EBV-specific CTLs to patients with advanced NPC is feasible, appears to be safe, and can be associated with significant antitumor activity (114).
**CTL following anti-CD45 mAb administration**	2009	8	1/8: CR; 2/8: SD; 5/8: PD; Lymphodepleting mAbs prior CTL transfer may represent an alternative to chemotherapy to enhance expansion of infused CTL (109).
**EBV-specific CTLs**	2010	23	Treatment of patients with relapsed/refractory EBV-positive NPC with EBV-CTLs is safe and can be associated with significant, long-term clinical benefit, particularly for patients with locoregional disease (108).
**Higher doses of Autologous EBV-specific CTLs(CTL following cyclophosphamide and fludarabine CT)**	2012	11	EBV-specific CTL therapy is safe and associated with antitumor activity in patients with advanced NPC;Preparative lymphodepleting chemotherapy does not improve clinical results (115).
**EBV-CTL following GC CT**	2014	38	3/38: CR; 22/38: PR; 11/38: SD; 1/38: PD; 1/38: N/A; 3-year OS: 37.1%; These study achieved one of the best survival outcomes in patients with advanced NPC, setting the stage for a future randomized study of chemotherapy with and without EBV-CTL (116).
**Autologous EBV-specific CTL**	2014	1	After infusion, the majority of pulmonary lesions were no longer evident, although the primary tumor did not regress (117).
**AdE1-LMPpoly vector-based CTL**	2017	29	Adoptive immunotherapy with AdE1-LMPpoly-expanded T cells stabilizes relapsed, refractory NPC without significant toxicity (118).

CR, complete response; CT, chemotherapy; CTL, cytotoxic T-cell; GC, gemcitabine and carboplatin; N/A, not available; ORR, objective response rate; OS, overall survival; PD, progressive disease; SD, stable disease.

Notably, the reaction rate varied in different clinical studies, primarily because these trials utilized different techniques to produce EBV-specific CTLs. In addition, these trials involved patients at different NPC stages, which could also have distinct previous treatments, comorbidities, and genetic susceptibility. To raise the immunogenicity and enhance antigen specificity, a new adenovirus vector, AdE1-LMPpoly was generated that encoded EBNA1, which covalently binds to multiple epitopes in CD8^+^ T cell (119). A clinical study showed that AdE1-LMPpoly vector stimulated CD8^+^ T cells in Hodgkin lymphoma donors, stimulating the rapid expansion of EBNA1 and LMP1/2-specific CD8^+^ T cells (120). Two studies using AdE1-LMPpoly confirmed that MVA vaccines harboring the EBNA1 sequence prevented and treated lymphoma (90, 121). A clinical trial to treat NPC by EBV-specific T cells induced by the new AdE1-LMPpoly vector showed that sixteen of 24 patients with NPC had expanded EBV-specific T cells, whereas no effect was noted in 27.3% of NPC cases. Furthermore, compared with the control group, the median overall survival increased from 220 days to 523 days (65). In subsequent studies, the same team demonstrated that T cell therapy combined with AdE1-LMPpoly was well tolerated in high-risk cases without residual diseases and cases with recurrent/metastatic diseases (111).

A phase 2 clinical trial was carried out by Chia et al. to assess the efficacy of chemotherapy combined with EBV-specific CTLs in 35 patients. The effective rate of combined therapy was 71.4%, with five cases not needing further chemotherapy over 34 months after the start of treatment with CTLs. Compared with all similar studies, this trial achieved the best results in patients with advanced NPC, which shows that chemotherapy combined with EBV-specific CTL therapy is a very promising approach (116).

### 3.2 EBV-specific chimeric antigen receptor T cell therapy

**Figure 4:**
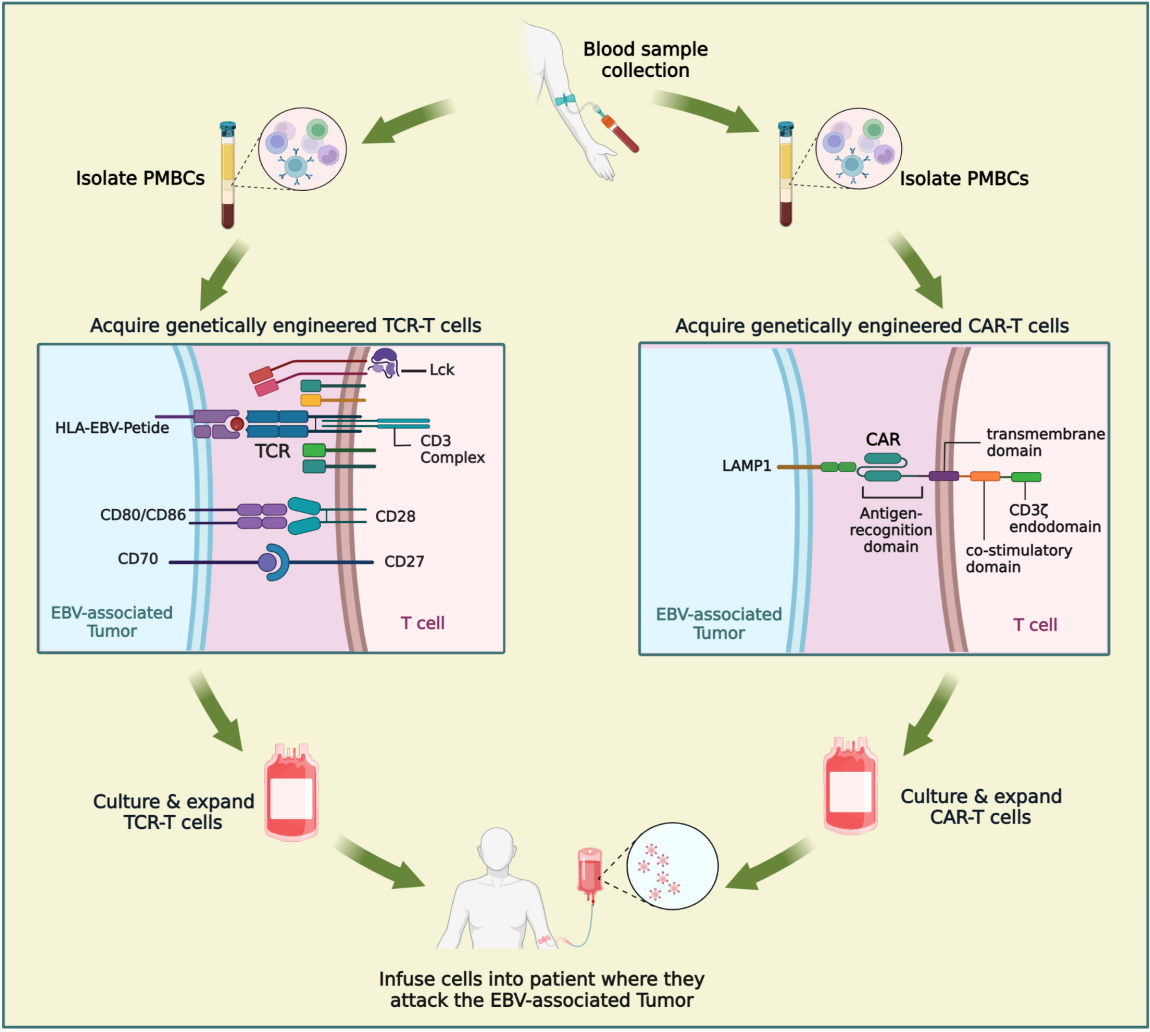
Comparison of CAR-T and TCR-T cells in the treatment of NPC. Engineered immune cell therapy works by modifying immune cells so that they can recognize disease states and respond appropriately. When engineered immune cells are transferred into patient, they are a “living drug”. TCR-T is similar to CAR-T in that it involves engineering a patient’s own T lymphocytes and then injecting back into the patient. The procedure mainly includes: extraction of patients’ peripheral blood, isolation of PBMC, engineering of immune cells, amplification of immune cells and cell transfusions into patients. Figure was created with BioRender.

Chimeric antigen receptor T cell immunotherapy (CAR-T) has been tested for many years but was only recently approved for humans. CAR-T has shown a good efficacy in treating to acute leukemia and non-Hodgkin lymphoma and is considered to be one of the most promising tumor treatment modalities (**Figure 4**). Currently, there are eight approved CAR-T products: six targeting CD19 and two targeting BCMA (B cell maturation antigen). CAR-T is applied primarily in hematological malignancies, such as diffuse large B cell lymphoma (DLBCL), B cell acute lymphoblastic leukemia (B-ALL) as well as recurrent or refractory forms of multiple myeloma, follicular lymphoma, and mantle cell lymphoma. Although the innovative immune therapy with CAR-T cells shows considerable efficacy in hematological malignancies, its application in solid tumors is difficult (122). Despite the efforts of scientists, clinical doctors, and pharmaceutical companies worldwide, CAR-T cells have not been clinically approved to treat any solid tumor. The lack of good target antigens is the greatest obstacles facing in the CAR-T therapies (123, 124). Encouragingly, there are more than 1,100 registered clinical trials evaluating the treatment of cancers using CAR-T cells (59 have been completed), of which approximately 200 relate to solid tumors.

As of today, six registered clinical studies are evaluating the efficacy of NPC by CAR-T therapies (**Table 3**). A phase 1 trial (NCT02915445) conducted on patients with NPC or breast cancer aimed to evaluate the therapy with CAR-T cells specifically targeting epithelial cell adhesion molecule (EpCAM). EpCAM-specific CAR-T cells were administered to different cohorts of patients (NCT02915445). *In vitro* assays demonstrated that LMP1-specific CAR-T cells kill 70% of NPC cells overexpressing LMP1. Furthermore, injecting LMP1-specific CAR-T cells into mice with tumors markedly reduced the number of LMP1^high^ NPC cells. Although it is doubtful that LMP1-specific CAR-T cells can target cancer cells in NPC with much lower LMP1 expression, these results are encouraging (125, 126), and a clinical trial for LMP1-specific CAR-T cells to treat EBV-associated malignant tumors is underway (NCT02980315). Furthermore, a phase I study designed to treat patients with relapsed or refractory NPC is ongoing (NCT04107142).

**Table 3:** Ongoing clinical trials of CAR-T and TCR-T in NPC.

Trial Number	Phase	Estimated enrollment	Study Title	Interwentions	Locations
NCT03013712	Phase 1/2	60	A Clinical Research of CAR T Cells Targeting EpCAM Positive Cancer	Biological: CAR-T cell immunotherapy	China
NCT02980315	Phase 1/2	20	A New EBV Related Technologies of T Cells in Treating Malignant Tumors and Clinical Application	CAR-T cell	China
NCT02915445	Phase 1	30	EpCAM CAR-T for Treatment of Advanced Solid Tumors	Biological: EpCAM CAR-T cells	China
NCT05239143	Phase 1	100	P-MUC1C-ALLO1 Allogeneic CAR-T Cells in the Treatment of Subjects with Advanced or Metastatic Solid Tumors	Biological: P-MUC1C-ALLO1 CAR-T cells Drug: Rimiducid	United States
NCT03648697	Phase 2	20	EBV-TCR-T(YT-E001)for Patients With EBV-positive Recurrent or Metastatic NPC	Biological: EBV-TCR-T (YT-E001) cells	China
NCT04107142	Phase 1	10	Haplo/Allogeneic NKG2DL-targeting Chimeric Antigen Receptor-grafted γδ T Cells for Relapsed or Refractory Solid Tumour	Biological: Adoptive Cell Transfer of NKG2DL-targetting Chimeric Antigen Receptor-grafted Gamma Delta T cell	Malaysia
NCT04509726	Phase 1/2	20	LMP2-Specific IL12-secreting TCR-T Cells in the Treatment of EBV-Positive Met	Drug: LMP2 Antigen-specific TCR T cells	China
NCT03648697	Phase 2	20	EBV-TCR-T(YT-E001)for Patients With EBV-positive Recurrent or Metastatic NPC	Biological: EBV-TCR-T (YT-E001) cells	China
NCT03925896	Phase 1	27	Phase I Trial of LMP2 Antigen-specific TCR T-cell Therapy for Recurrent and Metastatic NPC Patients	Drug: LMP2 Antigen-specific TCR T cells	China
NCT05587543	Phase 1	24	Clinical Study on the EBV CAR-T/TCR-T Cells in the Treatment of Nasopharyngeal Carcinoma	Behavioral: PK Blood Collection Drug: CAR, TCR	China

### 3.3 T cell receptor-engineered T cell therapy

T cell receptor-engineered T cell therapy (TCR-T) has quietly arrived on the cancer therapy scene (**Figure 4**). TCR-T has shown unprecedented potential in the therapy of solid tumors and has gradually attracted increasing attention because of its ability to target a variety of antigens within tumors. TCR-T is similar to CAR-T in that it involves engineering a patient’s own T lymphocytes and then injecting back into the patient. However, the mechanisms by which TCR-T and CAR-T recognize antigens differ. As an antigen recognition element in T cell therapy, TCR can recognize a wider range of potential tumor-specific antigens, especially the ultra-sensitive recognition of low-level variation of intracellular antigens, while CAR recognizes only tumor cell surface antigens. The immunosuppressive tumor microenvironment and tumor-associated antigen expression rate on the cell surface of solid tumors are low, and most of the cell proteins are intracellular. These factors limit the clinical application of CAR-T in solid tumors, while TCR-T may have a more effective role against solid tumors. In nearly two years, clinical studies of TCR-T cell therapy in HBV-related HCC and HPV-related cervical cancer have been reported, and good results have been achieved (127–129). There are more and more studies on the treatment of nasopharyngeal carcinoma by TCR-T cells. LMP2 antigens are a potential target of TCR-T cells; targeted clearance of cells containing LMP2 may have robust antitumor properties and limited toxicity to normal cells. Tumor progression in LMP2-expressing NPC cell lines implanted in mice was inhibited by LMP2-specific TCR-T cells (130). Similar results were observed for the LMP1-specific TCR-T cells (131). At present, there are four ongoing clinical trials evaluating TCR-T for NPC (NCT04509726, NCT03925896, NCT03648697, NCT05587543) (**Table 3**). The first clinical study of TCR-T therapy for NPC was launched in 2018, in which NPC patients with high expression of LMP1, LMP2, and EBNA1 were selected for study. The TCR targets were screened to explore the role of EBV antigen-specific T cells (YT-E001) in RM-NPC patients (NCT03648697). The latest clinical trial, first posted in October 2022, aims to compare the efficacy of CAR-T and TCR-T cells in the treatment of NPC (NCT05587543). TCR-T therapy for cancer is an exciting and rapidly developing field. The application of TCR-T therapy has pioneered an innovation h to treating cancer, viral infections, and other immunomodulatory diseases. It is hoped that more appropriate immune targets will be selected, and TCR-T transfection methods will be optimized in the near future through the unremitting efforts of researchers. TCR-T therapy is bound to play an significant role in cancer, infectious diseases, or autoimmune diseases.

### 3.4 NK cell therapy

Our team has been actively developing new approaches and targets for the treatment of EBV-NPC. We found that after EBV infection, LMP2A induced the upregulation of F3 expression and was associated with the dysfunction of NK cells in NPC (2). We used a combination of F3 inhibitor and NK cell adoptive therapy and achieved a considerable therapeutic effect in a mouse model of NPC. Another study by our group was performed to elucidate the mechanisms involved in EBV-induced NK cell dysfunction. We found that deletion of B7-H3 on tumor cells, in combination with anti-PD-L1(programmed death-ligand 1) treatment, restored NK cell-mediated antitumor activities and showed synergistic therapeutic efficacy. This study, which is currently under submission, aims to provide a rationale for NK cell-based immunotherapies in combination with PD-L1 blockade for overcoming the immunosuppression of B7-H3 to treat EBV-associated NPC. Therefore, targeting EBV-related signaling pathways, combined with NK cell adoptive therapy, may be a new direction to explore for the treatment of NPC in the future.

## 4 Immune checkpoint inhibitors

Patients with RM-NPC have limited treatment options, making chemotherapy the main treatment; however, its curative effect is unsatisfactory (34, 132–137). The emergence of immunotherapy has turned around the therapy for RM-NPC. Cisplatin combined with gemcitabine is the first-line therapy for RM-NPC; however, the second-line treatment is still lacking (138–140). Programmed death-1 (PD-1) and PD-L1 are associated with tumor immune escape and immunotherapy, which are critical for the tumor survival. NPC induced by EBV often presents with high levels of PD-L1 and substantial lymphocyte infiltration, thus, application of PD-1 blockade immunotherapy may be beneficial (141). Indeed, preclinical studies have shown that EBV proteins LMP1 and EBNA1/2 regulate PD-L1 levels, thereby modulating the extent of immune escape (142–144).

Treatment of NPC by immune checkpoint inhibitors (ICIs) is currently a hot research topic. Effective ICIs may break the immune defense and revitalize endogenous antitumor immunity (**Figure 3C**). Primary targets of ICIs include PD-1/L1 and cytotoxic T lymphocyte-associated protein 4 (CTLA-4). Three clinical trials of anti-PD-1 in patients with RM-NPC reported the objective response rates in the range of 20.5–34.1% (145–148). To date, a considerable number of ICIs have been licensed for use by the FDA.

Pembrolizumab has been demonstrated to have a good antitumor activity and safety profile in previously treated patients with RM-NPC (147, 149–151). Another ongoing phase II trial (NCT03544099) aims to evaluate the efficacy of pembrolizumab for NPC patients with plasma EBV DNA after radio-chemotherapy. A study of nivolumab in patients with RM-NPC reported promising antitumor activity and favorable 1-year overall survival rates (145). A case of NPC was reported in which the patient was given nivacizumab, a PD-L1 inhibitor, because of the high PD-L1 expression in his tumor. Following this treatment, it was found that the tumor rapidly and completely subsided, and there was no recurrence after 22 months of treatment. This study sets a foundation for future trials, involving many NPC patients (152). Fan et al. showed that camrelizumab, another anti-PD-1 inhibitor also had strong antitumor activity and was well tolerated in patients with RM-NPC. Based on the above results in these two papers, the authors will start a phase III trial to treat the patients with RM-NPC by using chemotherapy combined with PD-1 inhibitors (148). A phase 3 trial aimed to study the effect of RM-NPC treated by chemotherapy combined with camrelizumab in the treatment of RM-NPC. The results showed considerable prolongation of progression-free survival in patients that underwent combination therapy; therefore, the combination of camrelizumab and chemotherapy may become a new method to treat NPC (153).

On February 19, 2021, an anti-PD-1 monoclonal antibody named toripalimab was licensed by the National Medical Products Administration in China to treat the patients with RM-NPC whom had previously failed second-line or systemic therapy. Thus, toripalimab has become the first anti-PD-1 monoclonal antibody approved for NPC treatment in the world as a breakthrough in immunotherapy in this field (154). Wan et al. reported key clinical results of treatment with toripalizumab (POLARIS-02), which showed that the drug is safe, controllable, and induces a lasting clinical response in patients with refractory NPC that poorly respond to chemotherapy (155). More than 80 clinical studies aiming to evaluate anti-PD-1 treatments for NPC have been or are currently being conducted, with 20 of them testing the efficacy of toripalimab.

CTLA-4, belongs to the CD28 immunoglobulin subfamily and functions as a receptor that suppresses T cell activation (156). CTLA-4 and PD-1 are two of the earliest targets for which ICIs have been developed. The immune response rate to an ICI treatment is limited because it is a single-drug therapy, which, in turn, restricts the clinical application of ICIs (157). After years of clinical experience, the direction of CTLA-4 inhibitor development has become increasingly clear: the combination therapy of CTLA-4 inhibitor and PD-1 or PD-L1 may have the broadest application. CTLA-4 and PD-L1 work within different parts of the immune system and exert distinct effects. CTLA-4 acts primarily in the priming phase and is the main factor affecting antigen presentation. PD-L1 primarily acts in the effector phase, blocking immune checkpoints and inducing tumor death. Therefore, blocking both CTLA-4 and PD-L1 may enhance antitumor effects. Ipilimumab is the only CTLA-4 drugs authorized worldwide, furthermore, many anti-CTLA-4-targeting inhibitors in development and more than 20 in clinical trials. Ipilimumab is combined with pembrolizumab and nivolumab for immunotherapy of highly metastatic colorectal cancer, gastroesophageal, and other cancers (158–162). Combination therapy is more effective than conventional monoclonal antibody therapy and provides an opportunity for the development and application of bi-specific antibodies. The first PD-1/CTLA-4 bi-specific antibody product approved worldwide was developed by Akeso, Inc. The range of this antibody is applicable to a series of solid tumors, such as cervical cancer, lung cancer, gastric cancer, and NPC (163). Continuous monitoring of adverse events is required during treatment with bi-specific blockers, as inhibiting multiple targets simultaneously may increase the frequency of side effects.

Our team have found that anti-human CD39 antibody increased the expression of human CD8+T cells and inhibited human B-cell lymphoma after autologous EBV-specific T cell metastasis (164, 165). Based on our study, we conducted the first human trial of anti-CD39 in patients with advanced cancer (NCT03884556) (164, 165). These studies have provided new targets and ideas for the immunotherapy of EBV-NPC.

## 5 Conclusion

Tumor immunotherapy is a new technological approach that can overcome the disadvantages of the three traditional methods in cancer therapy: surgery, radiotherapy, and chemotherapy. In particular, tumor immunotherapy may provide a better outcome in cases of incompletely surgically removed, metastatic, and easy-to-relapse tumors. The specificity of immunotherapy effectively kills tumor cells in the absence of serious side effects and prolongs patient survival. Immunotherapy controls the proliferation of tumor cells by irritating and enhancing immune functions in endogenous organisms, and its current main approaches are based on the use of vaccination, ACT, and ICIs. At present, there are several therapeutic strategies to generate EBV vaccines, including the prevention of EBV infection, modulation of the incubation period, and inhibition of the occurrence of EBV-associated tumors. Development of the polymeric EBV vaccine containing gH/gL, gB, and gp350 is a particularly promising direction. Therapeutic vaccines against EBV are likely to be more efficient than preventive vaccines because it takes a long time for EBV infection to induce diseases; therefore, developing preventive vaccines against EBV-related diseases such as NPC may not be possible. Most therapeutic vaccine preparations currently use EBNA1, LMP1, and LMP2 as targets. These proteins play an important role in the malignant transformation of EBV-infected cells. During the last few years, many studies have achieved remarkable successes in developing ACT against EBV antigens. EBV vaccine together with T cell infusion may be a good way to treat NPC and this combination can enhance the specificity of T cells. To enhance the clinical efficacy of ACT therapy in NPC, CAR-T cells raised against an EBV target and a surface receptor may be utilized. To expand the efficiency of CAR-T cells, new antigen targets for EBV-induced NPC should be urgently identified. In recent years, many cellular immunotherapies have emerged and are developing rapidly, undoubtedly setting off an upsurge in cancer treatment. T cell immunotherapy in general is a hot field in cellular immunotherapy. Both CAR-T cells and TCR-T cells are T cells modified by genetic engineering. While TCR-T therapy may not be as well known to the general public, it may offer advantages over CAR-T therapy in the treatment of solid tumors. Another possible effective strategy to treat EBV-NPC is to combine ACT with ICIs against PD-1/L1, or CTLA-4. The optimization of these methods is expected to render immunotherapy the first-line therapy for various EBV-related diseases. Tumor immunotherapy also decreases the incidence of adverse reactions and complications arising from chemotherapy. It can kill two birds with one stone, thus protecting NPC patients. Therefore, chemotherapy combined with immunotherapy may increase the efficacy of the treatment and reduce its toxicity for NPC patients. Integrative therapy that combines NPC immunotherapy with conventional therapeutic approach is not simply combination of existing methods but, rather, a combination of treatments that achieves the highest synergy.

Although most immunotherapy strategies for EBV-related NPC are still at the clinical trial stage, the published and emerging research results indicate that immunotherapy for NPC may soon be used in clinical settings. Ongoing research hopes to achieve a selection of practical immune targets and optimized TCR-T transfection methods in the near future. We believe that TCR-T cell therapy may currently be the most promising approach to treatment of EBV.

## Author contributions

WL, XBD and XC performed literature review and wrote the manuscript. MZ and HP revised the figures and tables. YM and XL revised the manuscript. X-YL, GP, and XHD developed the review concept. All authors contributed to the article and approved the submitted version.
